# Supplementary figures and images for: DHEA Induces Sex-Associated Differential Patterns in Cytokine and Antibody Levels in Mice Infected with Plasmodium berghei ANKA
Source: Int J Mol Sci. 2023 Aug 8;24(16):12549. doi: 10.3390/ijms241612549 (PMC10454633; doi:10.3390/ijms241612549)

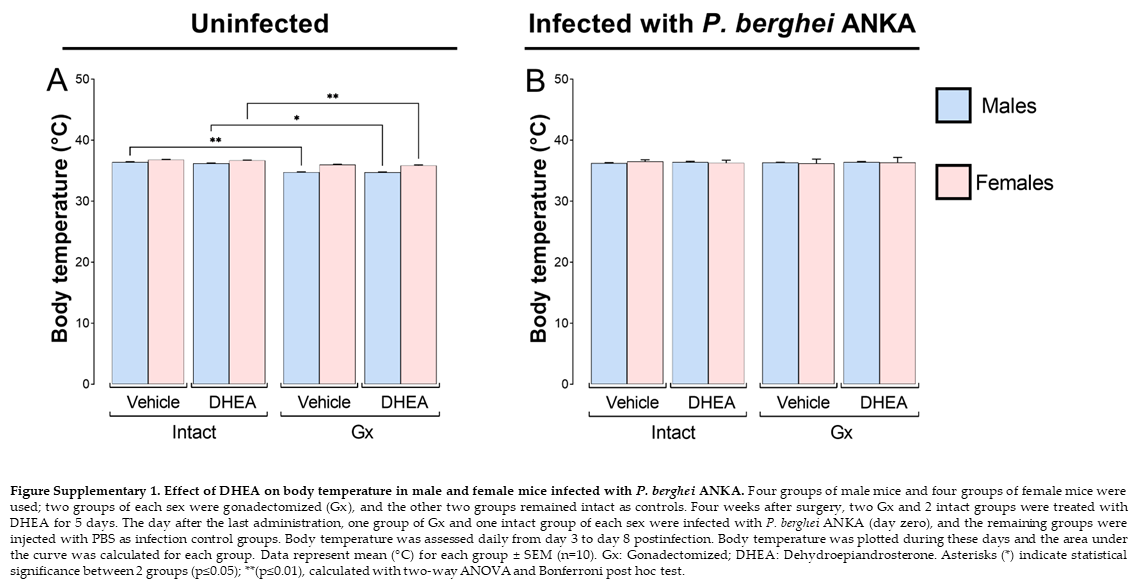

Supplement: Supplementary file 1 [file ijms-24-12549-s001.zip › Figure S1.tif]

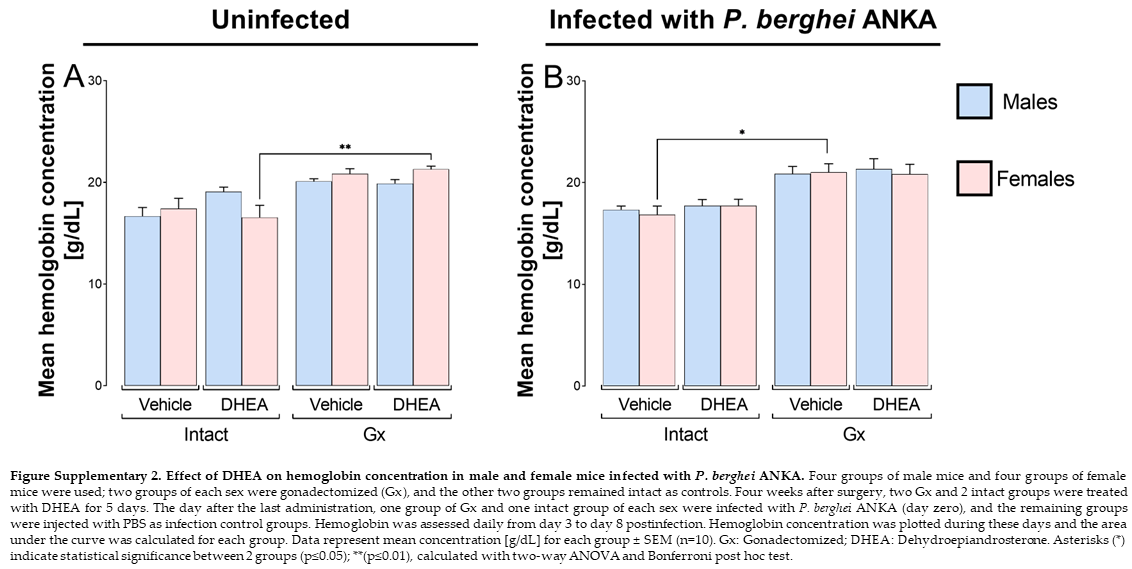

Supplement: Supplementary file 1 [file ijms-24-12549-s001.zip › Figure S2.tif]

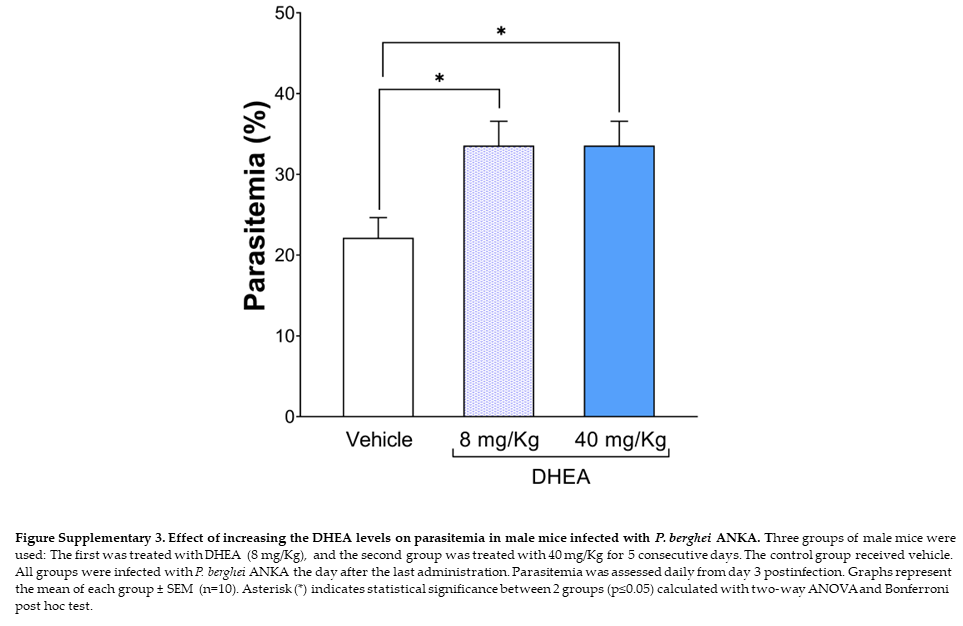

Supplement: Supplementary file 1 [file ijms-24-12549-s001.zip › Figure S3.tif]
